# A novel strategy for identifying hepatotoxic constituents in traditional Chinese medicine using dose-normalized intracellular accumulation as a cytotoxicity indicator: a case study of Jinlingzi San

**DOI:** 10.3389/fphar.2025.1585186

**Published:** 2025-05-30

**Authors:** Xiaoting Gu, Hanyang Wang, Keran Li, Cuiting Wu, Xin Di

**Affiliations:** ^1^ State Key Laboratory of Medicinal Chemical Biology, College of Pharmacy and Tianjin Key Laboratory of Molecular Drug Research, Nankai University, Tianjin, China; ^2^ Laboratory of Drug Metabolism and Pharmacokinetics, School of Pharmacy, Shenyang Pharmaceutical University, Shenyang, Liaoning, China; ^3^ Centre for Applied Pharmacokinetic Research, The University of Manchester, Manchester, United Kingdom

**Keywords:** traditional Chinese medicines, hepatotoxicity, dose-normalized intracellular accumulation, LC-MS/MS, Jinlingzi San

## Abstract

**Introduction:**

Herb-induced liver injury associated with traditional Chinese medicine (TCM) has increasingly attracted scientific attention. However, the rapid and effective methods for elucidating the material basis of hepatotoxicity in TCM are still lacking. This study developed a strategy based on the use of dose-normalized intracellular accumulation as a cytotoxicity indicator to identify key hepatotoxic components in TCM.

**Methods:**

Jinlingzi San (JLZS) composed of Fructus Toosendan (FT) and Rhizoma Corydalis (RC) was used as a model sample in this study. Hepatotoxicity was evaluated through both in vivo (14-day continuous gavage administration in rats) and in vitro (24-hour co-incubation with L-02 cells) models. Chemical components in JLZS extract and those accumulated in L-02 cells were identified using LC-MS/MS. The intracellular accumulations of multiple components after exposing L-02 cells to the extracts of JLZS, FT and RC were determined.

**Results:**

JLZS administration resulted in subacute liver injury in rats and demonstrated cytotoxicity to L-02 cells. Seven analytes (coptisine, tetrahydrocoptisine, berberine, jatrorrhizine, palmatine, dehydrocorydaline and toosendanin) were identified in cell lysates following incubation with JLZS. Coptisine and berberine were regarded as the main potential hepatotoxic components in JLZS for its highest dose-normalized intracellular accumulation and the strongest cytotoxicity. In addition, the intracellular accumulations of coptisine and berberine were lower in the JLZS group compared to the RC group alone.

**Discussion:**

The findings suggest that dose-normalized intracellular accumulation serves as a reliable indicator for identifying hepatotoxic constituents in TCM. The observed reduction in intracellular accumulation of coptisine and berberine in the JLZS formulation compared to RC alone maybe reflect the scientific meaning of detoxicity by compatibility with FT and RC.

## 1 Introduction

Herb-induced liver injury (HILI) associated with traditional Chinese medicine (TCM) has been widely reported and has garnered increasing attention (Zhang et al., 2024). HILI can be categorized into intrinsic HILI and idiosyncratic HILI, as defined in clinical hepatotoxicity studies (Teschke and Danan, 2021). Intrinsic HILI is predictable and dose dependent, typically exhibiting a short latency period. In contrast, idiosyncratic HILI is unpredictable and generally dose independent. The issue of causality assessment in HILI cases has been a subject of debate for a long time. The Roussel Uclaf Causality Assessment Method (RUCAM) has been established and validated in 1993 (Bénichou et al., 1993; Danan and Bénichou, 1993) and refined in 2016 (Danan and Teschke, 2016), and the updated RUCAM became the gold standard tool for causality assessment in HILI cases, particularly for complex TCM formulations. RUCAM had evaluated as many as 14,029 cases of HILI from 1993 until 30 June 2020, with the largest number, 11,609 cases, published in 2019 following the update of RUCAM and its ongoing worldwide use for assessing causality in suspected HILI cases, surpassing all other causality assessment methods in terms of case volume (Teschke and Danan, 2020). Recent studies have validated RUCAM in HILI cases. For instance, Xia et al. (2020) used the updated RUCAM to evaluate skyfruit-induced liver injury, confirming a highly probable causal relationship between the botanical drug and hepatotoxicity. Li et al. (2024) used the updated RUCAM and integrated evidence chain to evaluate Xianling Gubao-induced liver injury, confirming a highly probable causal relationship between the TCM and hepatotoxicity. These studies highlight RUCAM’s adaptability to TCM complexities, incorporating adjustments for polypharmacy risks and traditional usage patterns. Traditional approaches for identifying hepatotoxic components in TCM often focus on isolated compounds, which may overlook the synergistic effects of multicomponent formulations (Pan et al., 2023; Teka et al., 2021). However, clinical causality assessment (e.g., via RUCAM) must evaluate the entire herbal mixture as hepatotoxicity may arise from compound interactions rather than individual components (Zhou et al., 2021). This holistic approach is critical for accurate risk assessment in TCM. Therefore, there is a pressing need for more rapid and effective screening strategies that consider the interactions and combined effects of various constituents.

According to the theory that dose determines toxicity, intracellular concentration of a potential cytotoxic drug in target cells could effectively mediate its cytotoxic effects (Zhang et al., 2015; Schürmeyer et al., 2025). Our research group pioneered a novel whole-cell-based strategy to identify hepatotoxic components in TCM. This innovative approach used intracellular accumulation as a cytotoxicity indicator, enabling the effective and cost-efficient screening of hepatotoxic components in TCM. By applying this strategy, we successfully identified three potential hepatotoxic components in *Chelidonium majus* L (Wu C. T. et al., 2019). However, the concentration of a specific component in TCM extract can significantly affect its intracellular accumulation. Even if only a small portion of the component is taken up by cells, a high concentration in the extract may still lead to elevated intracellular levels. To address this issue, dose-normalized intracellular accumulation—defined as the ratio of a component’s intracellular concentration to its concentration in the medium—may be possible to provide a more reliable indicator of cytotoxicity as it more accurately reflects a component’s capacity for intracellular accumulation. In this study, the feasibility of using dose-normalized intracellular accumulation as a cytotoxicity indicator for screening hepatotoxic components in TCM was investigated for the first time.

Jinlingzi San (JLZS), composed of Fructus Toosendan (mature fruits of Melia toosendan Siebold and Zucc.*a synonym of *Melia azedarach* L. [Meliaceae]), FT) and equal amount of Rhizoma Corydalis (dry tubers of *Corydalis yanhusuo* W.T. Wang, RC), was reported to induce subacute toxicity through the disorder of energy metabolism and oxidative stress regulation (Shen et al., 2017). FT has frequently been reported to cause liver injury (Yu et al., 2020; Chang et al., 2023). As the main bioactive component in FT, toosendanin (TSN) could cause liver injury by inducing hepatocyte energy metabolism disorder and impairing DNA damage responses and autophagy (Yan et al., 2019; Yang et al., 2019; Luo et al., 2022; Lin et al., 2025). Furthermore, several studies have reported that RC can cause liver injury (Wang, 2021; Guo et al., 2025). RC is rich in isoquinoline alkaloids, which belong to the toxic secondary metabolites occurring in plants of many families (Anna et al., 2019; Dai et al., 2023). Isoquinoline alkaloids in RC can be divided into tertiary and quaternary alkaloids, according to the different charge properties of the nitrogen atom. The quaternary alkaloids such as coptisine (COP) and palmatine (PAL) could cause liver injury (Ma et al., 2010; Yi et al., 2013; Lu et al., 2023); a previous study has reported that berberine (BER) can directly inhibit hepatic gluconeogenesis by suppressing energy metabolism and pyruvate carboxylation, consequently inducing hepatotoxicity (Moreira et al., 2022).

Until now, the components responsible for the hepatotoxicity for JLZS remain unclear. Therefore, developing an effective method to identify the hepatotoxic components in JLZS is urgently needed. In the present study, the hepatotoxicity of JLZS on rats and L-02 cells was investigated at first. A fully validated, sensitive, and selective LC–MS/MS method was developed to determine the seven compounds of JLZS in cell lysates simultaneously. Afterward, the potential hepatotoxic compounds of JLZS were screened using the proposed LC–MS/MS method, based on a whole-cell strategy of dose-normalized intracellular accumulation as an indicator for cytotoxicity. Additionally, the intracellular accumulation compatibility study of FT and RC was conducted to elucidate the synergistic effects of JLZS. This study will provide important information on the hepatotoxic basis of JLZS and offer a systemic strategy for the identification of hepatotoxic components in TCM.

## 2 Materials and methods

### 2.1 Materials

FT and RC were bought from GuoDa Pharmacy (Shenyang, China). TSN, corydaline (COR), and protopine (PRO) standards were purchased from Chengdu Pufei De Biotech Co., Ltd (Sichuan, China). Tetrahydroberberine (THB), tetrahydrocoptisine (THC), jatrorrhizine (JAT), and dehydrocorydaline (DHC) standards were provided by Chengdu Herbpurify Co., Ltd (Sichuan, China). The reference standards of tetrahydropalmatine (THP), COP, berberine (BER), allocryptopine (ALL), and palmatine chloride were obtained from Chengdu MUST Bio-tech Co., Ltd. (Sichuan, China). Rutin (RUT) and diphenhydramine (IS) standards were provided by the National Institute for the Control of Pharmaceutical and Biological Products (Beijing, China). RPMI-1640 medium, trypsin-EDTA, Gemini Foundation™ fetal bovine serum, penicillin/streptomycin solution, PBS (−), and Cell Counting Kit-8 (CCK8) were purchased from Dalian Meilun Biotechnology Co., Ltd. (Dalian, China). Cell culture plates (9 cm-diameter) and 96-well cell culture plates were provided by Sangon Biotech Co., Ltd. (Shanghai, China). Dimethyl sulfoxide (DMSO) was provided by Damao chemical reagent factory (Tianjin, China). HPLC grade methanol and acetonitrile were bought from Concord Technology Co., Ltd. (Tianjin, China). Formic acid (HPLC grade) was provided by DIKMA technologies Inc. (Lake Forest, CA, USA). Deionized water was obtained from Wahaha Corporation (Hangzhou, China) and was used throughout.

### 2.2 Experimental animal

Sixteen male Sprague–Dawley rats (weigh 180–220 g) were purchased from Liaoning Changsheng Biotechnology Co., Ltd. The animal experiment program was approved by the Animal Ethics Committee of Shenyang Pharmaceutical University. Before experiment, rats were randomly divided into two groups (n = 8): the blank control group (BCG) and the JLZS group.

### 2.3 Animal treatments

A total of 1,000 g of JLZS powders were refluxed twice with 5,000 mL of 50% ethanol for 2 h. The filtrates were evaporated, vacuum-dried, and finally suspended into 0.5% sodium carboxymethyl cellulose (CMC-Na) to obtain the JLZS extracts.

To investigate the hepatotoxicity caused by JLZS, rats were perfused with 20.25 g/kg (calculated as raw botanical drug) of JLZS extracts in the JLZS group and equal volume of saline in the BCG once a day for 14 consecutive days. The dosing of JLZS was set according to the dose range reported in a previous study on the hepatotoxicity of JLZS in rats (Shen et al., 2017). Over the duration of the experiment, the general states of the rats were closely monitored, and the weights of rats were recorded. Furthermore, the blood samples (0.3 mL) were collected and kept at room temperature for 30 min and then centrifuged at 3,500 g for 10 min at 4°C to obtain supernatant serum. The activity of serum ALT or AST was tested using commercial kits. On the last day of the experiment, the rats were decapitated, and the liver tissues were collected. After fixing a piece of liver in 10% neutral formaldehyde solution and then embedding in paraffin wax, the liver was stained with hematoxylin and eosin (HE) for liver histopathological examination.

### 2.4 Cell culture

Human normal liver L-02 cells, offered by the Cell Bank of the Chinese Academy of Sciences (Shanghai, China), were grown in RPMI-1640 containing 10% fetal bovine serum and 100 U/mL penicillin/streptomycin at 37°C in a humidified atmosphere of 95% air–5% CO_2_.

### 2.5 Preparation of cell samples

The powder of 25 g FT and 25 g RC was evenly blended and then refluxed twice with six-fold 50% ethanol (1: 6, *w/v*) for 1 h; the collected filtrate was evaporated to dryness and further dissolved in DMSO, after filtering using the 0.22-µm cellulose acetate membrane. JLZS-containing medium (6.5 mg/mL) was freshly prepared by diluting the filtrate with RPMI-1640. Likewise, RC-containing medium was freshly prepared in the same method independently.

COR, THP, THB, THC, JAT, COP, BER, DHC, PRO, PAL, ALL, TSN, and RUT standards were weighed accurately and dissolved in DMSO to prepare the sample stock solution at the concentration of 10 mg/mL. After filtering using the 0.22-µm cellulose acetate membrane, the sterile stock solutions of compounds were serially diluted with RPMI-1640 to test its cytotoxicity.

### 2.6 Preparation of calibration standards and QC samples

After weighting and dissolving standard in methanol/water (50:50, *v/v*), the individual stock solution of TSN, COP, BER, JAT, PAL, DHC, and THC was mixed in appropriated aliquots to prepare a mixed stock solution with the concentration of TSN at 400 ng/mL, COP at 1,280 ng/mL, BER at 160 ng/mL, JAT at 640 ng/mL, PAL at 400 ng/mL, DHC 400 ng/mL, and THC at 640 ng/mL. Six series of mixed standard solutions were prepared by diluting the mixed stock solution serially. The concentration of the IS solution was 150 ng/mL. Quality control (QC) solutions (at low, medium, and high final concentrations) were prepared from an additional mixed stock solution freshly.

By spiking serial mixed standard solutions into blank cell lysates, the calibration standards and QC samples were prepared freshly.

### 2.7 Intracellular identification

L-02 cells were seeded on 9-cm-diameter cell culture plates; after ∼80% confluent, the cultured cells were incubated with 6.5 mg/mL JLZS-containing medium. After 2-h exposure, L-02 cells were quickly washed free of culture medium with cold PBS (−) twice and harvested with 1 mL of 0.25% trypsin-EDTA for cell isolation. The cell suspension (∼5 × 10^6^ cells) was spun down, and the cells were further washed twice in cold PBS (−). Then, 200 µL of methanol was used to lyse the collected cells with vortex mixing for 1 min. The cell lysates were centrifuged at 12,000 rpm for 4 min, and 10 μL of the centrifugal supernatant was injected into the LC–MS/MS system for identified analysis.

### 2.8 Intracellular accumulation

L-02 cells were grown as described in the intracellular identification study. Then, the cultured cells were randomly divided into the JLZS group and the RC group. After incubating with JLZS-containing medium (6.5 mg/mL, calculated as raw botanical drug) and RC-containing medium (3.25 mg/mL) for 0.25, 0.5, 1, 1.5, 2, 3, 5, and 8 h with six duplicates for each point, L-02 cells were washed and lysed according to the procedure described in the intracellular identification study. To determine the intracellular accumulation for test compounds, 20 μL cell lysates was mixed with 20 μL IS solution and 100 μL methanol with vortex mixing for 1 min; a measure of 5 μL of mixtures was used for the quantitative determination. Intracellular accumulations of analytes between different groups were compared using SPSS (version 19.0, IBM, USA) in one-way analysis of variance. *P* < 0.05 was considered to be statistically significant.

### 2.9 Instrument conditions for LC–MS/MS analysis

The LC–MS/MS system consisted of a Thermo TSQ Quantum Ultra triple-quadrupole mass spectrometer equipped with an electrospray ionization source (San Jose, CA, United States). LCquan quantitation software (version 2.5.6, Thermo Fisher Scientific, Waltham, MA, USA) was used to control the LC–MS/MS system for data acquisition and analysis. Analytes and IS separation were conducted using a Waters XTerra^®^ MS C18 column (3.0 mm × 50 mm, 5 µm) maintained at 20 °C. By injecting 5 µL samples with a flow rate of 0.2 mL/min, chromatographic separation was achieved using a mobile phase of 0.1% formic acid–water (A) and methanol (B) *via* gradient elution, 0.0–2.0 min, 35%–50% B; 2.0–2.1 min, 50%–55% B; 2.1–2.5 min, 55%–60% B; 2.5–14.0 min, 60% B. The analytes and IS were detected in the ESI source by polarity switching from positive for 0.0–8.0 min to negative-ion modes for 8.5–14.0 min in a single run with multiple reaction monitoring (MRM). The ion transitions and collision energies are listed in Table 1.

**TABLE 1:** MRM transitions and collision energies for the determination of analytes and IS.

Compound	Precursor ion (*m/z*)	Product ion (*m/z*)	Collision energy (eV)	ESI
TSN	573	531	23	−
COP	320	292	37	+
THC	324	176	24	+
BER	336	320	27	+
JAT	338	322	27	+
PAL	352	336	29	+
DHC	366	350	30	+
IS	256	167	25	+

### 2.10 Cell growth inhibition assay

L-02 cells were plated in 96-well plates (15,000 cells/well) in the volume of 100 µL. After plating for 24 h, the medium was replaced with the fresh medium containing test samples at series concentrations. Cell growth inhibition was assessed 24 h after exposure to the test samples at 37°C using the CCK-8 assay, according to the manufacturer’s protocol. In brief, 10 µL of WST-8 solution was diluted with RPMI-1640 at a ratio of 1:9 and added into each well of 96-well plates at 37°C for 2 h. The absorbance was detected at a wavelength of 450 nm using a microplate reader (Corona Electric, Ibaraki-Ken, Japan). IC_50_ values were calculated using Graphpad Prism 5.0 (GraphPad software Inc., San Diego, CA).

## 3 Results

### 3.1 The hepatotoxicity of JLZS on rats

**FIGURE 1:**
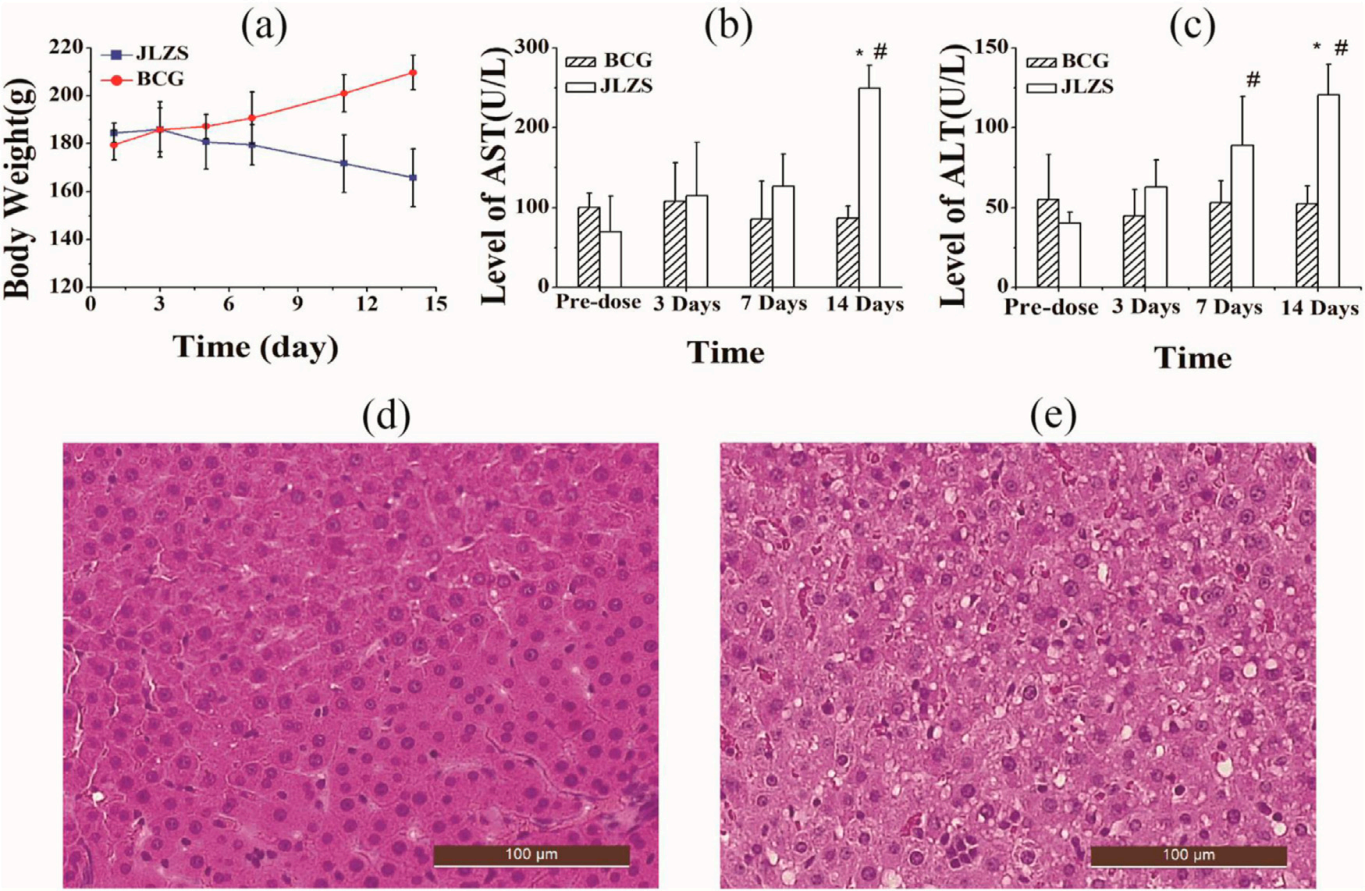
Subacute liver injury caused by JLZS. **(a)** Body weights of the rats in the JLZS group and BCG, AST value **(b)**, and ALT value **(c)** in rat serum of pre-dose and 3 days, 7 days, and 14 days post-dose (*, *p* < 0.05, compared with BCG; #, *p* < 0.05, compared with pre-dose); histopathological photographs of rat livers in the BCG **(d)** and JLZS **(e)** group. Data were expressed as mean ± SD (n = 8).

During the period of experiment, the rats in the BCG showed good mental state, whereas the rats in the JLZS group suffered from mental depression, insensitive erect fur, anorexia, and fear of cold. Body weights of the rats in different groups are displayed in Figure 1a. The weights of rats increased gradually in the BCG but decreased progressively in the JLZS group during the 14-day administration, and significant difference was observed since the third day. The significant difference from the two different groups indicated that JLZS has certain toxicity on the whole level of rats.

Serum transaminase ALT and AST that mainly existed in hepatocytes are golden standard biomarkers for evaluating the liver function. Liver function injury can induce the increase in the permeability of cell membrane and is always accompanied by the increase in ALT and AST levels released from the cytoplasm into the serum. In this study, the contents of AST and ALT in the serum of rats for different groups are displayed in Figures 1b,c. Compared with the BCG, it can be observed that both the serum ALT and AST in the JLZS group were increased significantly (*p* < 0.05) at 14 days after oral administration of JLZS extracts. Contemporaneously, the levels of serum ALT and AST in the JLZS group were increased progressively with the increase in administration times and showed significant difference (*p* < 0.05) at the end of the trial compared with pre-dose. These phenomena implied that there might be a substantial lesion for the liver of rats.

Histopathological photographs of liver tissues in different groups are shown in Figures 1d,e. In the BCG, the liver tissue was stained evenly, the cell edge was clear, and the cell arranged regularly. On the contrary, the hallmarks of cell boundary fuzzy, cell swelling, the nucleus shrinkage, cytoplasmatic vacuoles, and widespread hydropic degeneration of hepatocytes were observed from the histopathology in the JLZS group, suggesting serious liver injury caused by JLZS extracts after repeated administration. Therefore, JLZS could cause obvious liver injury on rats after oral administration of JLZS extracts with large doses repeatedly.

### 3.2 Identification of L-02 cell-uptake components in JLZS

**FIGURE 2:**
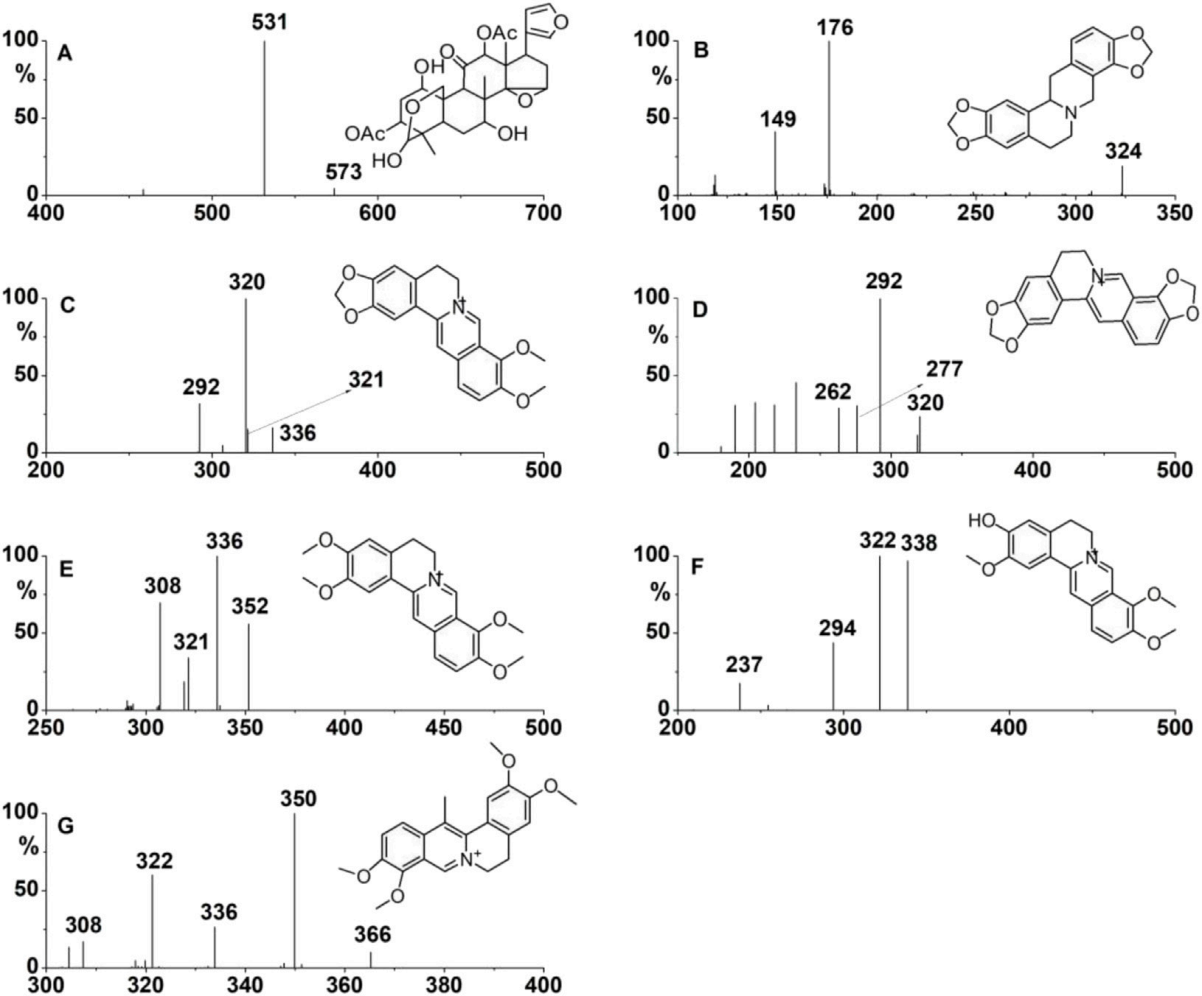
Product ion spectra and chemical structures of TSN **(A)**, THC **(B)**, BER **(C)**, COP **(D)**, PAL **(E)**, JAT **(F)**, and DHC **(G)**.

**FIGURE 3:**
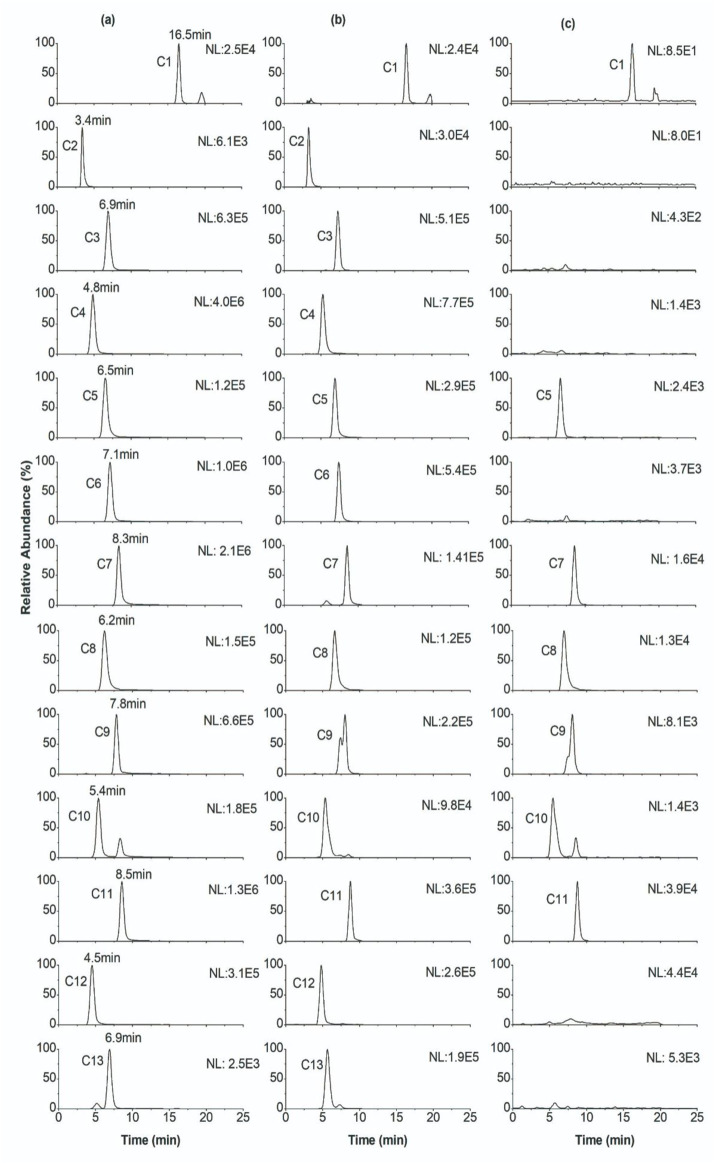
Product ion scan chromatograms of standard substances **(a)**, extracts of JLZS **(b)**, and cell lysates after exposing to JLZS for 2 h **(c)**.

Thirteen components (COR, THP, THB, THC, JAT, COP, BER, DHC, PRO, PAL, ALL, TSN, and RUT) were identified in the extract of JLZS by comparing their MS splitting decomposition law and retention time with those acquired from the reference substances. The MS^2^ fragment ions and retention time of thirteen components are summarized in Table 2. After exposing L-02 with JLZS-containing medium (6.5 mg/mL) for 2 h, TSN and all quaternary alkaloids of isoquinoline alkaloids (COP, BER, PAL, JAT, and DHC) but only one tertiary alkaloid (THC) were found in the cell lysates. Product ion spectra and chemical structures of these components are displayed in Figure 2. Product ion scan chromatograms of standard substances, extracts of JLZS, and cell lysates after exposing to JLZS for 2 h are displayed in Figure 3.

**TABLE 2:** Retention time (t_R_) and MS^2^ fragment ions of thirteen components identified in JLZS.

Peak no.	Compounds	Formular	t_R_ (min)	MS	MS^2^
C1	TSN	C_30_H_38_O_11_	16.5, 19.5	573	531
C2	RUT	C_27_H_30_O_16_	3.4	609	300, 271, 178
C3	COR	C_22_H_27_NO_4_	6.9	370	352, 303, 219, 205, 192, 163
C4	THP	C_21_H_25_NO_4_	4.8	356	192, 165, 151
C5	THC	C_19_H_17_NO_4_	6.5	324	176, 174, 149
C6	THB	C_20_H_21_NO_4_	7.1	340	176, 149
C7	BER	C_20_H_18_NO_4_	8.3	336	321, 320, 304, 292
C8	COP	C_19_H_14_NO_4_	6.2	320	292, 277, 262, 249
C9	PAL	C_21_H_22_NO_4_	7.8	352	336, 308
C10	JAT	C_20_H_20_NO_4_	5.4	338	322, 294, 237
C11	DHC	C_22_H_24_NO_4_	8.5	366	350, 336, 322, 308
C12	PRO	C_20_H_20_NO_5_	4.5	354	336, 305, 275, 247, 206, 188
C13	ALL	C_21_H_24_NO_5_	6.9	370	352, 336, 306, 290, 188

### 3.3 Intracellular accumulation of L-02 cell-uptake components in JLZS

**FIGURE 4:**
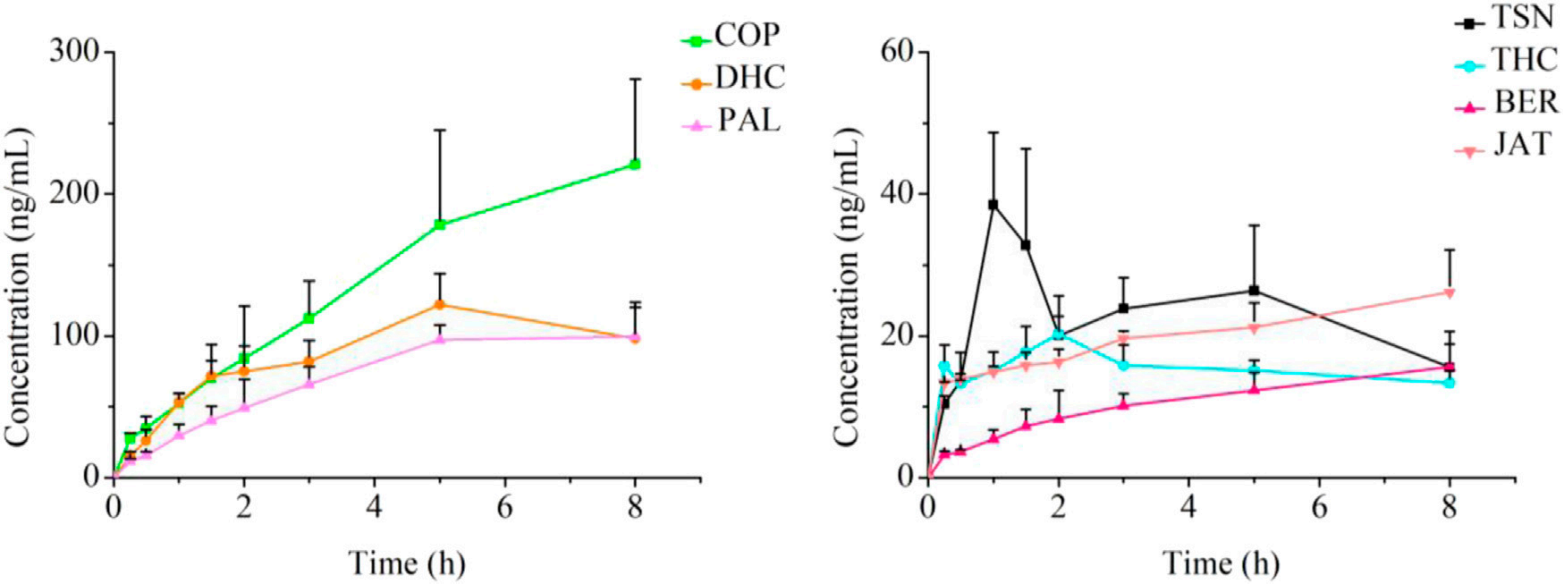
Concentration curves of seven analytes in L-02 cells after co-incubation with JLZS extracts (n = 6).

The intracellular concentrations of the seven tested substances in the JLZS group, RC group, and FT group at tested time points were determined through LC–MS/MS. The results of the LC–MS/MS method validation are presented in the Supplementary Material. The intracellular concentration of seven analytes after co-incubation with JLZS extracts are summarized in Table 3. With the exposure time increasing, the concentration–time curves of the seven analytes in cell lysates are displayed in Figure 4, showing that the intracellular accumulations of analytes increased progressively with the incubation time and finally reached a plateau. The intracellular accumulation of COP was the highest, followed by DHC and PAL, those of which were significantly higher than that of other alkaloids. The intracellular accumulation of TSN reached a highest concentration after exposing to JLZS for 1 h but decreased quickly to a plateau.

**TABLE 3:** Intracellular concentration of seven analytes after co-incubation with JLZS extracts (n = 6).

Time (h)	Concentration (ng/mL)						
	TSN	COP	THC	BER	JAT	PAL	DHC
0	0	0	0	0	0	0	0
0.25	10.4 ± 1.1	38.3 ± 4.9	15.8 ± 3.0	3.2 ± 0.5	13.4 ± 0.2	11.6 ± 1.8	15.2 ± 2.9
0.5	13.6 ± 4.0	44.0 ± 11.4	13.3 ± 0.5	3.7 ± 0.3	13.8 ± 0.8	15.6 ± 2.7	26.1 ± 3.0
1	38.5 ± 10.2	67.5 ± 7.7	15.0 ± 2.7	5.5 ± 1.3	14.9 ± 0.8	29.6 ± 7.9	53.1 ± 4.0
1.5	32.8 ± 13.6	84.4 ± 21.2	17.7 ± 3.6	7.2 ± 2.5	15.8 ± 1.8	40.2 ± 10.1	71.9 ± 5.5
2	20.1 ± 2.8	98.1 ± 34.8	20.3 ± 5.4	8.3 ± 4.0	16.3 ± 1.9	49.4 ± 19.9	74.8 ± 6.2
3	23.8 ± 4.4	134.8 ± 24.4	15.8 ± 2.9	10.1 ± 1.7	19.6 ± 1.0	65.7 ± 12.4	82.0 ± 7.5
5	26.4 ± 9.2	197.6 ± 45.9	15.1 ± 1.4	12.3 ± 2.5	21.2 ± 3.5	96.9 ± 10.7	122.0 ± 8.7
8	15.5 ± 3.4	261.8 ± 60.2	13.3 ± 1.8	15.6 ± 4.9	26.2 ± 6.0	99.5 ± 24.3	98.2 ± 9.6

### 3.4 Dose-normalized intracellular accumulation of L-02 cell-uptake components in JLZS

**FIGURE 5:**
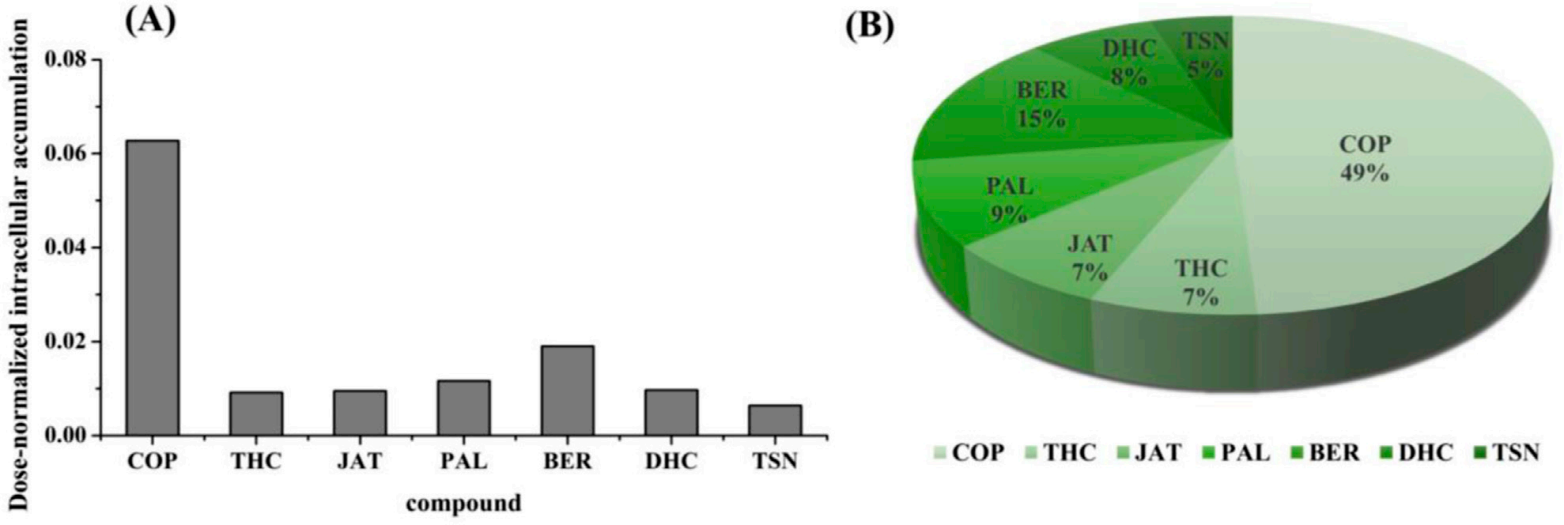
**(A)** Dose-normalized intracellular accumulation of analytes and **(B)** the proportion of dose-normalized intracellular accumulation for analytes.

The content of the identified compounds in JLZS varied. Intracellular accumulation without considering content could not accurately indicate the capacity of compounds entering cells. Dose-normalized intracellular accumulation was the ratio of the highest intracellular accumulation of compound versus its content in JLZS extracts. The concentration of the identified compounds in JLZS-containing medium was quantitatively determined using the developed LC−MS/MS method. The contents of COP, THC, JAT, PAL, BER, DHC, and TSN in the JLZS-containing medium of every cell culture plate were 4.17, 2.21, 2.76, 8.55, 0.82, 12.57, and 6.04 µg, respectively. Dose-normalized intracellular accumulations of these identified compounds in JLZS are shown in Figure 5. It could be observed that the dose-normalized intracellular accumulation of COP was the highest, followed by BER, intracellular accumulation of which was higher than that of other compounds; however, the contents of COP and BER were not very high in JLZS. The ranking of dose-normalized intracellular accumulations of compounds indicated that COP and BER may be the main potential hepatotoxic compounds in JLZS.

### 3.5 Cell viability assay of main components in JLZS

**FIGURE 6:**
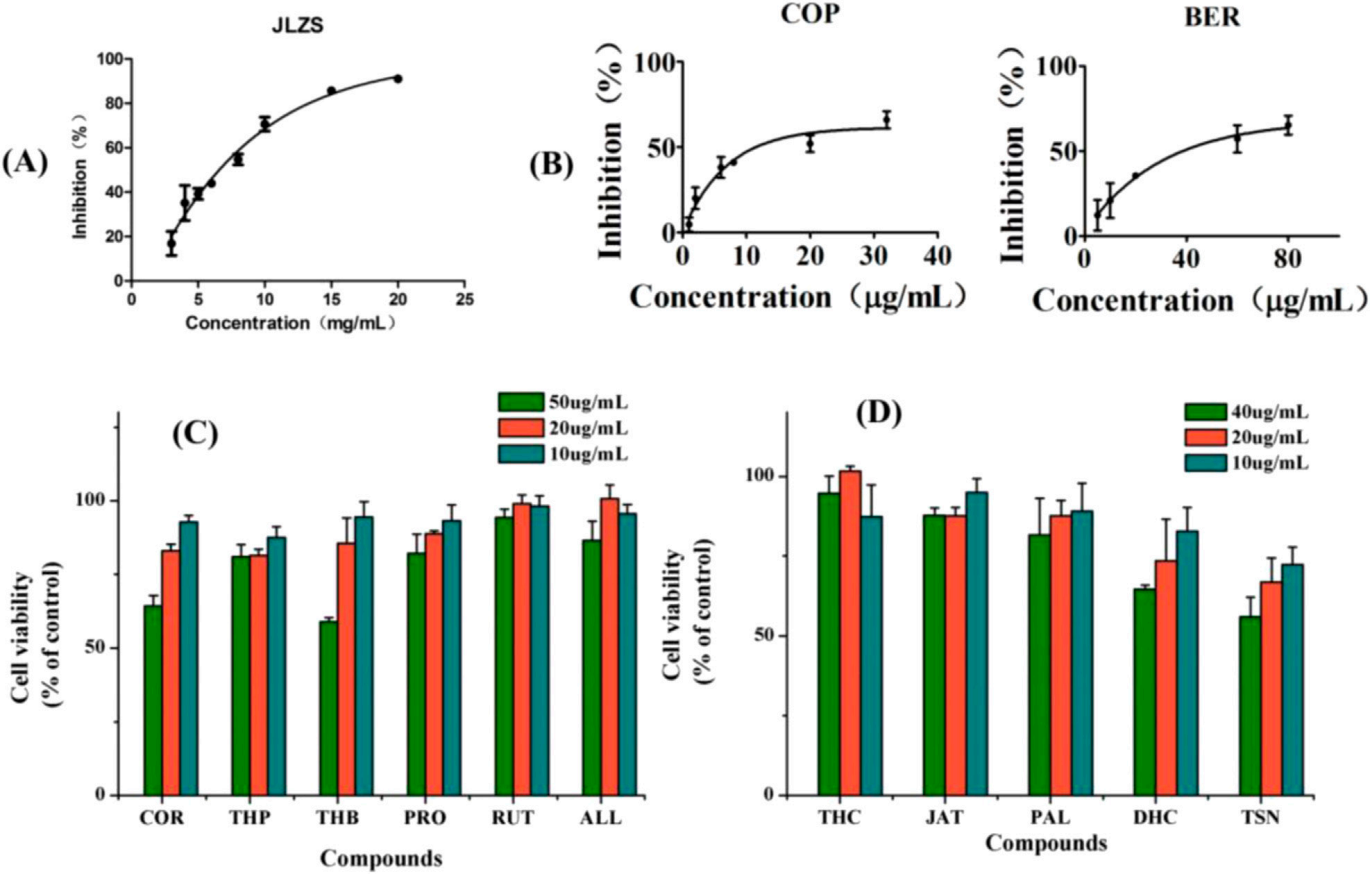
Inhibition rate of the cell viability to the JLZS concentration curve **(A)**, cell viability of compounds (n = 3): compounds with high dose-normalized intracellular accumulation **(B)**, compounds that do not enter L-02 **(C)**, and compounds with small dose-normalized intracellular accumulation **(D)**.

CCK-8 assay was used to test the effect of JLZS extracts and the identified compounds in JLZS on the survival rate of L-02. The IC_50_ values were calculated using GraphPad prism 5.0 software. The inhibition rate of the cell viability to the JLZS concentration curve is displayed in Figure 6A. The IC_50_ value of JLZS was 6.5 mg/mL. Cell viabilities of the identified compounds in JLZS are shown in Figures 6B–D. Among the identified compounds, COR, THP, THB, PRO, RUT, ALL, THC, JAT, PAL TSN, and DHC were not detected IC_50_ values within the set concentration range. Nevertheless, the IC_50_ values of COP and BER were 14.0 and 41.2 μg/mL, respectively, indicating that COP and BER have stronger hepatotoxicity than other compounds in JLZS. COP possesses the lowest IC_50_ values but highest dose-normalized intracellular accumulation.

### 3.6 Intracellular accumulation compatibility study of JLZS

**FIGURE 7:**
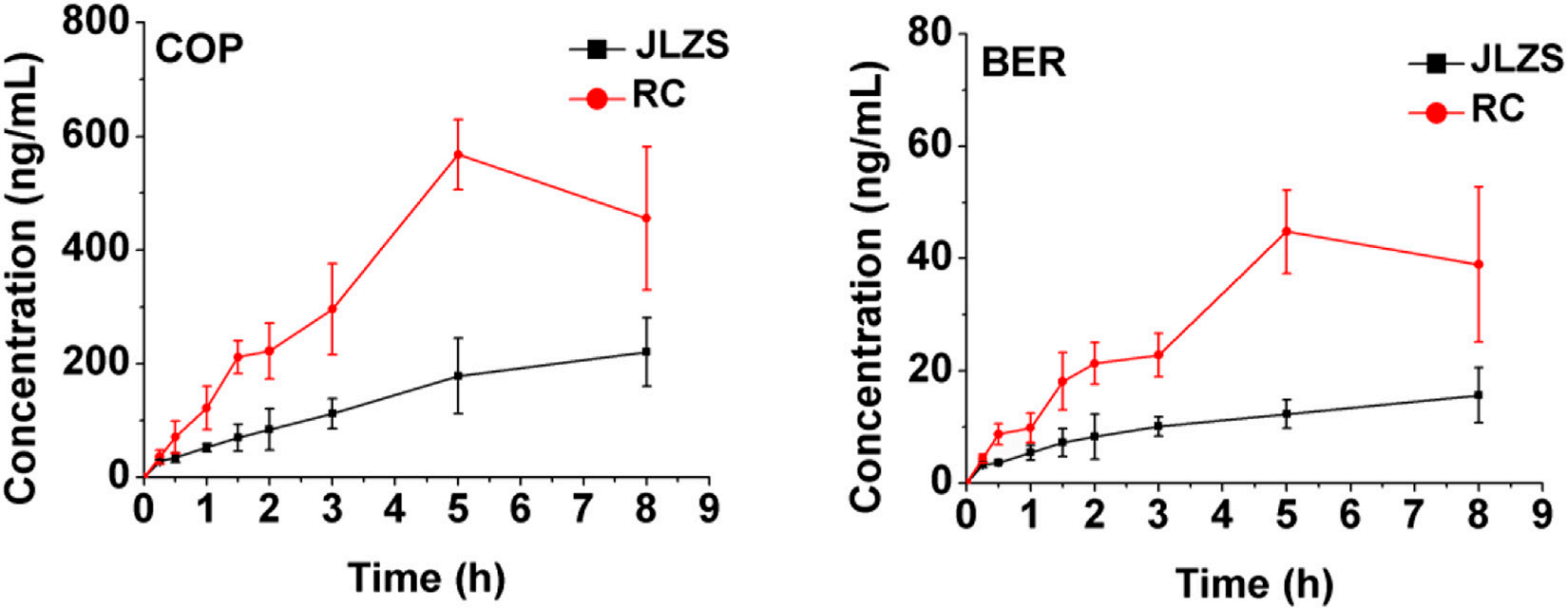
Intracellular concentration of COP and BER after co-incubation with JLZS and RC extracts (n = 6).

The intracellular accumulation of COP and BER in the JLZS group and RC group are displayed in Figure 7. The uptakes of COP and BER in the JLZS group were decreased significantly compared with the RC group. The reduction in the intracellular accumulation of the potential toxic components in JLZS reflected the scientific meaning of compatibility with FT and RC.

## 4 Discussion

As TCMs are increasingly widely used and monitoring technologies are continuously improved (He et al., 2018), reports of adverse reactions to botanical drugs, primarily centered around HILI, are becoming more frequent. This poses significant challenges to the development of new drugs and the clinical application of TCMs. For instance, the U.S. FDA has ordered a ban on the import and sale of TCMs containing the aristolochic acids due to its significant liver toxicity (Zhang et al., 2022). The hepatotoxic mechanisms caused by TCMs have not been fully elucidated. The pathogenesis of HILI might be related to oxidative stress, inflammatory responses, cell apoptosis, mitochondrial dysfunction, lipid metabolism, and the release of fibrosis-promoting factors (Zhang et al., 2024; Gu et al., 2025). Furthermore, the complexity arises from the interactions between multidrug combinations in TCMs (Zhou et al., 2021), which made the research on liver damage caused by TCMs more challenging and intricate. Currently, although the hepatotoxicity of FT has been reported (Yu et al., 2020; Chang et al., 2023), there is no systematic study on the hepatotoxicity of JLZS, which brings considerable uncertainty to its clinical application. In this study, JLZS was found to cause subacute liver injury in rats and cytotoxicity in L-02 cells for the first time, providing important reference data for its safe clinical use and medication management, such as monitoring patients’ liver function during clinical treatment and considering drug interactions when multiple drugs are used together.

Thirteen components (COR, THP, THB, THC, JAT, COP, BER, DHC, PRO, PAL, ALL, TSN, and RUT) were identified in the extract of JLZS. TSN, all quaternary alkaloids of isoquinoline alkaloids (COP, BER, PAL, JAT, and DHC), and only one tertiary alkaloid (THC) were found in the cell lysates. Notably, this study focused on intrinsic HILI (dose-dependent), where hepatotoxicity is predictable and directly correlates with the concentration of hepatotoxic components. COP and BER were identified as the main potential hepatotoxic compounds in JLZS using the proposed LC–MS/MS method based on the whole cell with the strategy of dose-normalized intracellular accumulation as an indicator for cytotoxicity. There was an interesting phenomenon that the toxicity of quaternary amines in isoquinoline alkaloids was generally stronger than that of tertiary amines. Cell viability of the 11 isoquinoline alkaloids in JLZS was analyzed in this study, including five quaternary alkaloids (COP, BER, PAL, JAT, and DHC) and six tertiary alkaloids (COR, THP, THB, PRO, ALL, and THC). From the results, none of six tertiary alkaloids demonstrated measurable IC_50_ values within the set concentration range. Nevertheless, COP and BER exhibited significant cytotoxicity with IC_50_ values of 14.0 and 41.2 μg/mL, respectively. Previous studies have reported that quaternary alkaloids such as COP, BER, and PAL could cause liver injury (Ma et al., 2010; Yi et al., 2013; Lu et al., 2023; Moreira et al., 2022). Xiang et al. (2024) reviewed the gastrointestinal protective effects, pharmacokinetics, and toxicity of protoberberine alkaloids, and reported the hepatocytotoxicity of BER, PAL, and berberrubine, all of which are quaternary alkaloids. This collective evidence supported our observation that the toxicity of quaternary alkaloids was generally stronger than that of tertiary alkaloids. Furthermore, quaternary alkaloids were more likely to enter L-02 than tertiary alkaloids. We generally assume that quaternary amines, due to their charged nature, were less likely to permeate cell membranes than tertiary amines. For example, BER could be metabolized into dihydroberberine by nitroreductase, and dihydroberberine has stronger lipophilicity and better permeability across cell membranes (Feng et al., 2015). In this paper, there may be some unexplored correlations between the strong accumulation ability of quaternary amines in L-02 cells and its cytotoxicity, which is worthy of further study. Furthermore, hepatocytes, being enriched with metabolic enzymes, serve as the principal site for compound biotransformation. Following hepatic metabolism, COP undergoes extensive biotransformation, including hydroxylation, dehydrogenation, demethylation, and glucuronidation (Wu J. S. et al., 2019). Similarly, BER is metabolized through phase I reactions (primarily demethylation), followed by phase II conjugation (glucuronidation or sulfation) (Haque et al., 2025; Xiang et al., 2024). A critical question requiring further investigation is whether the observed hepatotoxic effects stem from the parent compounds (COP and BER) or their metabolic derivatives. In future studies, further analysis of the *in vitro* and *in vivo* metabolites of COP and BER, along with comparison of the hepatotoxicity of the parent compounds and their metabolites, could significantly advance our understanding of JLZS toxicity mechanisms and provide valuable insights for safety evaluation of related compounds.

JLZS is composed of equal proportions of FT and RC, first mentioned in “Suwen Bing Ji Bao Ming Ji” written by Wansu Liu. It is a classical prescription for regulating Qi to relieve pain. In terms of pharmacological effects, FT and RC in the formulation of JLZS exhibited a synergistic effect; the analgesic and anti-inflammatory effects of JLZS were enhanced compared to single herbs (Wang et al., 2018). Additionally, synergic effects of combining FT with RC were observed at the pharmacokinetic level in our previous study (Gu et al., 2021). The elimination of TSN, four tertiary alkaloids (COR, THP, THC, and THB), and two quaternary alkaloids (PAL and DHC) decreased significantly in the JLZS group compared with the single-herb group. The AUC_0-t_ of the tertiary alkaloids in the JLZS group showed an increasing trend compared with that of the RC group. In this paper, the intracellular accumulation compatibility study revealed that the intracellular accumulations of the potential hepatotoxic components in toxic target cells in JLZS were decreased compared with the FT group and RC group significantly. The reduction in the intracellular accumulation of the potential hepatotoxic components in JLZS reflected the scientific meaning of detoxicity by compatibility with FT and RC.

## 5 Conclusion

JLZS could cause subacute liver injury in rats and cytotoxicity in L-02 cells. A systemic strategy using dose-normalized intracellular accumulation as an indicator of cytotoxicity to screen hepatotoxic compounds in JLZS has been successfully developed. A sensitive LC-MS/MS method was developed and fully verified to simultaneously determine seven compounds in cell lysates for the first time. COP and BER were screened as the main hepatotoxic compounds in JLZS based on their minimum IC_50_ values and high dose-normalized intracellular accumulation. This study provided information on the hepatotoxic basis of JLZS which would be helpful to clarify the hepatotoxic mechanisms of JLZS, and offered a systemic strategy for the identification of hepatotoxic compounds from TCMs rapidly. The study explored the relationship between differences in IC_50_ values and the corresponding differences in dose-normalized intracellular accumulations for various chemical components, highlighting the effectiveness of using dose-normalized intracellular accumulations as an indicator of hepatotoxicity.

## Data Availability

The original contributions presented in the study are included in the article/Supplementary Material; further inquiries can be directed to the corresponding authors.

## Abbreviations

HILI, herb-induced liver injury; TCM, traditional Chinese medicine; JLZS, Jinlingzi San; FT, Fructus Toosendan; RC, Rhizoma Corydalis; TSN, toosendanin; COR, corydaline; THP, tetrahydropalmatine; THC, tetrahydrocoptisine; THB, tetrahydroberberine; COP, coptisine; PAL, palmatine; DHC, dehydrocorydaline; JAT, jatrorrhizine; BER, berberine; PRO, protopine; ALL, allocryptopine; RUT, rutin; IS, internal standard; QC, quality control; LLOQ, lower limit of quantification; ULOQ, upper limit of quantification; MRM, multiple reaction monitoring; RE, relative error; RSD, relative standard deviation.
