# Supplementary material for: A novel strategy for identifying hepatotoxic constituents in traditional Chinese medicine using dose-normalized intracellular accumulation as a cytotoxicity indicator: a case study of Jinlingzi San
Source: Front Pharmacol. 2025 May 30;16:1585186. doi: 10.3389/fphar.2025.1585186 (PMC12162926; doi:10.3389/fphar.2025.1585186)
Supplement: Supplementary file 1 [file DataSheet1.docx]

***1.Method validation***

Validation of the method, including selectivity, carryover effect, crosstalk, linearity, lower limit of quantification (LLOQ), upper limit of quantification (ULOQ), precision, accuracy, matrix effect, recovery, and stability, was conducted in accordance with the FDA Bioanalytical Method Validation Guidance for Industry.

Six calibration standards were freshly prepared via spiking the mixed standard solution into blank rat plasma to yield the concentration range of 10.0-400 ng/mL for TSN, PAL and DHC, 32.0-1280 ng/mL for COP, 4.00 -160 ng/mL for BER, 16.0 - 640 ng/mL for JAT and THC. Similarly, quality control (QC) samples were freshly prepared at 3, 80, 300 ng/mL for TSN, PAL and DHC; 96, 256, 960 ng/mL for COP; 12, 32, 120 ng/mL for BER; 48, 128, 480 ng/mL for JAT and THC, respectively.

*1.1 Selectivity and Crosstalk Effect*

The selectivity was evaluated by analyzing the endogenous interferences in blank cell lysates. The peak area of TSN was calculated by the sum of the two chromatographic peaks with the retention time at 9.51 and 10.15 min because of its equilibrium tautomerism. The retention time of COP, THC, BER, JAT, PAL, DHC and IS were 4.17, 4.81, 6.19, 5.31, 6.03, 6.71 and 7.35 min, respectively. As shown in **Fig S1**, no significant interference was observed at the retention time of the analytes and IS which suggesting good selectivity of the method.

Crosstalk was evaluated following individual injections at a high concentration, and no cross-talk effect was observed.


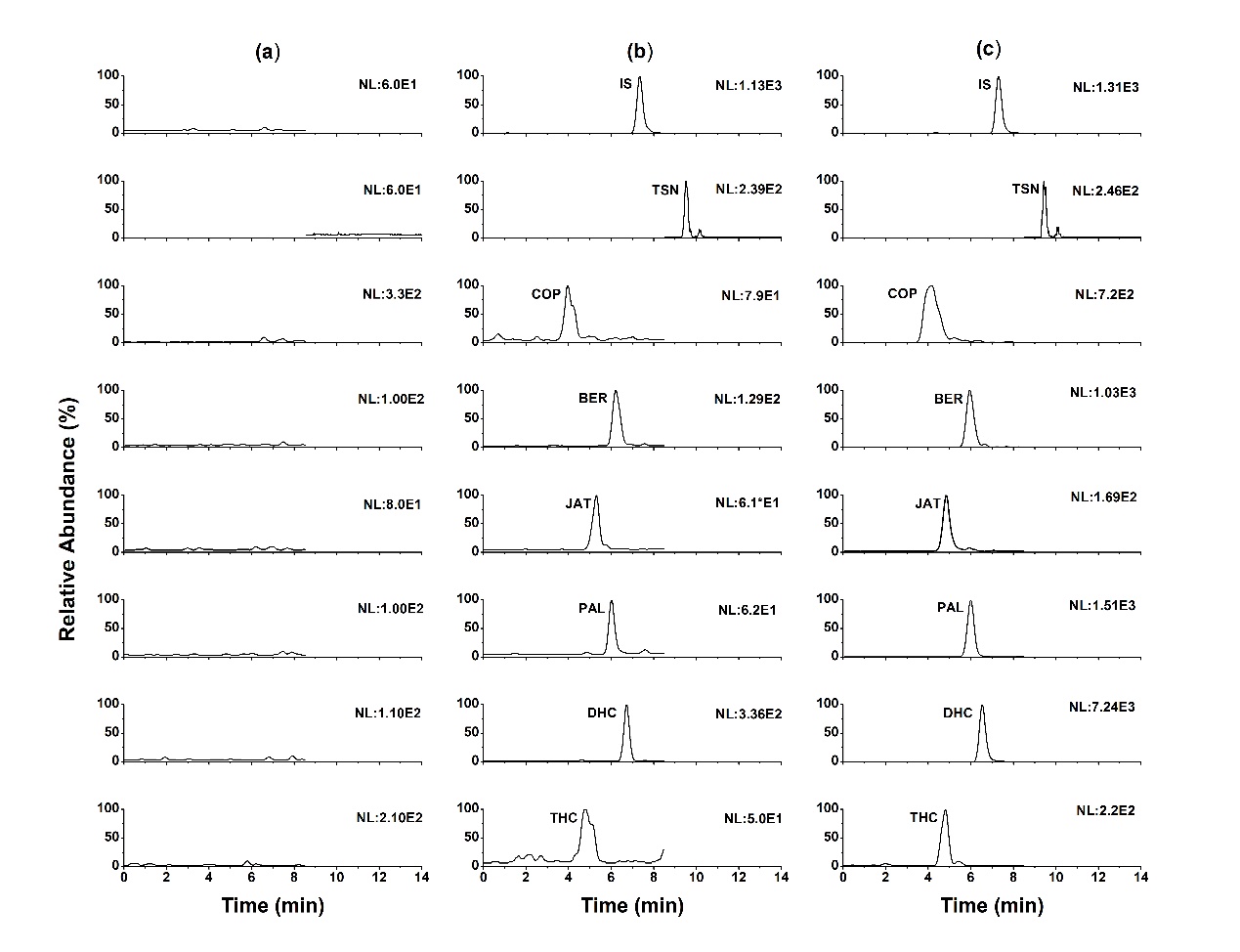


**Fig S1.** Typical chromatograms of seven analytes and IS: blank cell lysates (a); blank cell lysates spiked with seven analytes at LLOQ and IS (b) and cell lysates samples at 1 h after coincubation with JLZS (c).

*1.2 Linearity, LLOQs, and ULOQs*

Calibration curve was acquired by plotting the peak-area ratio of analyte to IS versus the nominal concentration and supplemented with least-squares linear regression analyses (1/x^2^ as a weighing factor). The precision and accuracy of LLOQs and ULOQs were estimated from by six replicates at the lowest and highest concentration respectively. The linear regression, LLOQ, ULOQ were summarized in **Table S1**. The calibration curves showed good linearity with correlation coefficients (r) greater than 0.9920. The LLOQs and ULOQs were acceptable by presenting adequate precision (RSD in the range of 1.6 % to 7.9 % at LLOQ and 2.1 % to 7.2 % at ULOQ) and accuracy (RE in the range of -9.7 % to 9.6 % at LLOQ; and RSD in the range of -0.4 % to 9.5 % at ULOQ) respectively.

**Table S1.** The linearity, LLOQ and ULOQ of seven analytes in cell lysates.

| compounds | Regression equation | r | Linear range  (ng/mL) | LLOQ | | | ULOQ | | |
| --- | --- | --- | --- | --- | --- | --- | --- | --- | --- |
|  |  |  |  | Nominal concentration (ng/mL) | RSD  % | RE  % | Nominal concentration (ng/mL) | RSD% | RE% |
| TSN | y=-0.00011+0.00001x | 0.9955 | 10–400 | 10 | 10.4 | -3.5 | 400 | 1.5 | 0.6 |
| COP | y = 0.00092+0.00080x | 0.9967 | 32–1280 | 32 | 4.1 | -7.8 | 1280 | 2.4 | 3.3 |
| BER | y = 0.00820+0.00488x | 0.9969 | 4–160 | 4 | 5.6 | -9.1 | 160 | 1.6 | 3.5 |
| JAT | y =0.01529+0.00496x | 0.9959 | 16–640 | 16 | 3.0 | -14.4 | 640 | 2.3 | 1.1 |
| PAL | y= 0.03728+0.00479x | 0.9932 | 10–400 | 10 | 2.5 | -16.2 | 400 | 1.9 | 0.9 |
| DHC | y=0.04994+0.00906x | 0.9953 | 10–400 | 10 | 2.8 | -17.9 | 400 | 5.7 | 1.9 |
| THC | y = 0.00470+0.00462x | 0.9979 | 16–640 | 16 | 3.5 | -9.4 | 640 | 2.5 | -3.5 |

*1.3 Accuracy and Precision*

By analyzing six replicates at three QC levels on one day or three validation days, the intra-day and inter-day precision and accuracy calculated to be satisfactory which were listed in **Table S2**. The imprecision and inaccuracy were below 15 % respectively.

**Table S2.** Precision and accuracy of analytes in cell lysates by LC–MS/MS analysis (n=6).

| Analytes | Theorical Concentration (ng/mL) | Intra-day | | | Inter-day | | |
| --- | --- | --- | --- | --- | --- | --- | --- |
|  |  | Measured concentration (ng/mL) | RSD (%) | RE (%) | Measured concentration (ng/mL) | RSD (%) | RE (%) |
| TSN | 30 | 29.02 | 7.68 | -3.28 | 31.15 | 14.78 | 3.82 |
|  | 80 | 82.36 | 6.01 | 2.95 | 84.64 | 7.14 | 5.80 |
|  | 300 | 322.45 | 4.03 | 7.48 | 328.81 | 4.42 | 9.60 |
| COP | 96 | 92.47 | 6.30 | -3.67 | 96.04 | 13.68 | 0.04 |
|  | 256 | 244.52 | 6.32 | -4.49 | 245.29 | 13.49 | -4.18 |
|  | 960 | 1016.38 | 4.53 | 5.87 | 1034.27 | 11.29 | 7.74 |
| BER | 12 | 12.33 | 6.99 | 2.71 | 12.03 | 14.73 | 0.26 |
|  | 32 | 32.10 | 5.46 | 0.30 | 32.00 | 14.29 | 0.01 |
|  | 120 | 117.41 | 2.85 | -2.16 | 124.87 | 13.27 | 4.06 |
| JAT | 48 | 47.13 | 5.84 | -1.82 | 47.02 | 13.87 | -2.03 |
|  | 128 | 128.71 | 4.73 | 0.56 | 127.56 | 14.04 | -0.34 |
|  | 480 | 515.20 | 3.19 | 7.33 | 523.07 | 7.90 | 8.97 |
| PAL | 30 | 27.04 | 5.12 | -9.87 | 28.65 | 14.09 | -4.49 |
|  | 80 | 73.70 | 3.00 | -7.87 | 75.71 | 13.02 | -5.36 |
|  | 300 | 270.90 | 4.60 | -9.70 | 288.38 | 12.99 | -3.87 |
| DHC | 30 | 29.89 | 3.87 | -0.35 | 30.87 | 11.22 | 2.91 |
|  | 80 | 79.78 | 4.10 | -0.28 | 80.29 | -13.94 | 0.36 |
|  | 300 | 258.89 | 3.38 | -13.70 | 271.98 | 12.20 | -9.34 |
| THC | 48 | 46.71 | 5.97 | -2.68 | 46.40 | 13.95 | -3.34 |
|  | 128 | 125.32 | 4.14 | -2.10 | 120.44 | 14.13 | -5.91 |
|  | 480 | 494.91 | 3.30 | 3.11 | 485.08 | 12.66 | 1.06 |

*RE-relative error; RSD-relative standard deviation.*

*1.4 Recovery and Matrix Effect*

The extraction recovery and matrix effect were investigated by analyzing six replicates at three QC levels. The results of extraction recovery and the matrix effect were summarized in **Table S3**. The mean extraction recoveries indicating extraction efficiency were more than 85.5 % (RSD ≤ 14.9 %) for all the analytes and 89.2 % for IS. The mean matrix effects ranged from 85.1 % to 111.5 % (RSD ≤ 14.9 %).

**Table S3.** Extraction recovery and matrix effect of analytes and IS in cell lysates by LC–MS/MS analysis (n=6).

| Compounds | Nominal concentration (ng/mL) | Extraction recovery | |  | Matrix effect | |
| --- | --- | --- | --- | --- | --- | --- |
|  |  | Mean (%) | RSD (%) |  | Mean (%) | RSD (%) |
| TSN | 30 | 89.0 | 8.9 |  | 104.8 | 12.5 |
|  | 80 | 90.4 | 9.5 |  | 88.4 | 13.4 |
|  | 300 | 85.2 | 3.9 |  | 85.7 | 8.4 |
| COP | 96 | 98.7 | 10.0 |  | 111.4 | 12.3 |
|  | 256 | 94.3 | 12.6 |  | 85.1 | 10.2 |
|  | 960 | 90.8 | 12.5 |  | 108.9 | 10.4 |
| BER | 12 | 114.7 | 8.9 |  | 104.4 | 12.0 |
|  | 32 | 87.2 | 12.2 |  | 106.5 | 13.6 |
|  | 120 | 93.5 | 7.2 |  | 92.9 | 10.1 |
| JAT | 48 | 89.7 | 13.1 |  | 108.8 | 6.3 |
|  | 128 | 85.5 | 11.6 |  | 87.4 | 11.9 |
|  | 480 | 110.2 | 13.5 |  | 106.4 | 5.5 |
| PAL | 30 | 86.1 | 9.8 |  | 96.8 | 13.9 |
|  | 80 | 89.8 | 14.9 |  | 85.9 | 12.5 |
|  | 300 | 102.6 | 11.8 |  | 105.5 | 13.2 |
| DHC | 30 | 112.8 | 13.8 |  | 111.5 | 12.6 |
|  | 80 | 90.0 | 12.9 |  | 90.0 | 14.2 |
|  | 300 | 100.7 | 11.0 |  | 111.2 | 8.9 |
| THC | 48 | 108.1 | 10.8 |  | 89.8 | 12.6 |
|  | 128 | 93.4 | 14.6 |  | 89.7 | 14.9 |
|  | 480 | 109.5 | 13.5 |  | 108.0 | 13.0 |
| IS | 150 | 89.2 | 11.8 |  | 106.5 | 13.8 |

*1.5 Stability*

The stability of seven analytes including short-term stability (12 h at room temperature), post-preparation stability at 4 ℃ (24 h at autosampler), freeze-thaw stability (three freeze-thaw stability) and long-term stability (-80℃ for a month) was tested by analyzing three replicates at low and high QC levels during various sample storage and handling process. The results of the stability of analytes were listed in **Table S4** which demonstrated that the analytes were stable during various sample storage and handling process.

**Table S4.** Stability of seven analytes and IS in cell lysates under various conditions (n=3).

| Analytes | Nominal concentration (ng/mL) | Stability | | | | | | | | | | |
| --- | --- | --- | --- | --- | --- | --- | --- | --- | --- | --- | --- | --- |
|  |  | Short-term | |  | Post-preparation | |  | Freeze-thaw | |  | Long-term | |
|  |  | RE% | RSD% |  | RE% | RSD% |  | RE% | RSD% |  | RE% | RSD% |
| TSN | 30 | 8.0 | 5.0 |  | 7.6 | 3.4 |  | 6.2 | 9.7 |  | 10.2 | 3.9 |
|  | 300 | -0.1 | 3.9 |  | 0.1 | 6.0 |  | -4.0 | 0.7 |  | 8.6 | 2.1 |
| COP | 96 | 5.6 | 8.0 |  | -0.6 | 10.0 |  | 0.7 | 10.1 |  | 4.0 | 8.6 |
|  | 960 | 6.2 | 4.1 |  | 6.8 | 5.5 |  | 5.0 | 6.5 |  | 6.6 | 8.8 |
| BER | 12 | 5.6 | 5.5 |  | 1.8 | 9.3 |  | 5.6 | 7.5 |  | 1.8 | 6.7 |
|  | 120 | 2.5 | 4.7 |  | 3.1 | 4.1 |  | 6.9 | 0.9 |  | 1.2 | 2.6 |
| JAT | 48 | 5.3 | 2.9 |  | -0.2 | 10.3 |  | 2.9 | 4.2 |  | 1.9 | 5.7 |
|  | 480 | 8.0 | 5.3 |  | 4.7 | 4.3 |  | 8.0 | 4.3 |  | 4.0 | 2.1 |
| PAL | 30 | 6.5 | 4.9 |  | -1.3 | 11.6 |  | -3.5 | 3.1 |  | 6.4 | 6.0 |
|  | 300 | 2.5 | 3.9 |  | 6.8 | 3.5 |  | 6.5 | 2.4 |  | 0.0 | 2.3 |
| DHC | 30 | -2.4 | 5.1 |  | -0.2 | 2.5 |  | -0.6 | 2.2 |  | 2.3 | 1.6 |
|  | 300 | 8.8 | 6.7 |  | 6.6 | 2.0 |  | 0.4 | 1.9 |  | 1.3 | 5.8 |
| THC | 48 | 3.4 | 9.1 |  | -0.6 | 10.1 |  | -2.5 | 10.6 |  | 5.3 | 6.1 |
|  | 480 | 4.6 | 8.0 |  | -0.9 | 7.6 |  | -1.4 | 6.2 |  | 1.2 | 8.4 |
